# Postoperative Complications of Free Flap Reconstruction in Moderate-Advanced Head and Neck Squamous Cell Carcinoma: A Prospective Cohort Study Based on Real-World Data

**DOI:** 10.3389/fonc.2022.792462

**Published:** 2022-06-24

**Authors:** Delong Li, Chong Wang, Wei Wei, Bo Li, Huan Liu, Aoming Cheng, Qifang Niu, Zhengxue Han, Zhien Feng

**Affiliations:** ^1^Department of Oral and Maxillofacial-Head and Neck Oncology, Beijing Stomatological Hospital, Capital Medical University, Beijing, China; ^2^Clinical Epidemiology and EBM Unit, National Clinical Research Center for Digestive Diseases, Beijing Friendship Hospital, Capital Medical University, Beijing, China

**Keywords:** postoperative complications, head and neck cancer, free flap reconstruction, risk factors, prediction model

## Abstract

**Background:**

Postoperative complications (POCs) of moderate-advanced head and neck squamous cell carcinoma (HNSCC) after free flap reconstruction have received little attention. We investigated the risk factors that lead to POCs and their impact on management and prognosis.

**Patients and Methods:**

A single-center, prospective cohort study was conducted at Beijing Stomatological Hospital on primary HNSCC patients treated between 2015 and 2020.

**Results:**

In total, 399 consecutive HNSCC patients who underwent radical resection of the primary tumor and free flap reconstruction were enrolled in this study, 155(38.8%) experienced POCs. The occurrence of POCs directly led to worse short-term outcomes and poorer long-term overall survival (P=0.0056). Weight loss before the operation (P=0.097), Tumor site (P=0.002), stage T4b (P=0.016), an ACE-27 index of 2-3 (P=0.040), operation time≥8h (P=0.001) and Clindamycin as antibiotic prophylaxis (P=0.001) were significantly associated with POCs.

**Conclusions:**

The occurrence of POCs significantly leads to worse short-term outcomes and increases the patients’ burden.

## Introduction

The long-term survival of patients with moderate-advanced head and neck squamous cell carcinoma (HNSCC) has not improved significantly in the past 40 years (1). During this period, surgery has remained the most common and effective treatment for primary HNSCC (2). Radical resection for moderate-advanced HNSCC involves large-scale tumor resection and neck dissection (ND), and some procedures involve free flap reconstructions and tracheostomy (3, 4). Even if the defect is repaired and reconstructed intraoperatively, a decline or loss of important functions and aesthetics is common after a major operation (2, 5).

Postoperative complications (POCs) as the most important reason for surgical failure, not only increase the patient’s health and economic burden, delay adjuvant treatment, and reduce short-term or long-term quality of life but also increase the risk of sequelae and a poor prognosis. Therefore, the prevention and management of surgical complications is becoming an issue that deserves more attention (6–8). It is important for HNSCC patients who undergo free flap reconstruction to obtain primary recovery without any POC, which may have a significant correlation with short-term outcomes and long-term survival (9, 10).

Many valuable works had been done about POCs of free-flap reconstruction for head and neck cancers, and reported rates of POCs ranged from 15% to 62% in published studies (10–15). Most of the literatures are retrospective study based on medical records or public database, while the occurrence and severity of complications were usually not defined or described so clearly (10–13). Despite many indexes, including the Frailty index (16), Kaplan-Feinstein score comorbidity index (17), Washington University Head and Neck Comorbidity Index (WUHNCI) (12), and Adult Comorbidity Evaluation-27 (ACE-27) (18), have been used to evaluate the preoperative status of populations with head and neck cancers and demonstrated to be related to increased risks of complications and decreased survival rates with increasing index scores, standardized methods for risk prediction developed specifically for POCs of HNSCC surgery with free flap reconstruction were still in need.

This prospective study was designed to investigate predictors of POCs occurring after HNSCC surgery with free flap reconstruction and their influence on survival in a real-world setting. Specifically, we sought to better characterize short-term and long-term outcomes after HNSCC surgery with free flap reconstruction and evaluate whether specific patient characteristics would be predictive of treatment effects, with the goal of providing useful guidance for clinical decision making.

## Patients and Methods

### Datasets

The data used in this study originated from POROMS, a Prospective, Observational, Real-world Oral Malignant Tumors Study (ClinicalTrials.gov identifier: NCT02395367). Chinese patients with newly diagnosed and pathologically confirmed stage II-IV HNSCC (UICC/AJCC classification 8^th^ edition) were treated in the Department of Oral and Maxillofacial‐Head and Neck Oncology, Beijing Stomatological Hospital, Capital Medical University, between March 2015 and May 2020. This prospective study was carried out in accordance with ethical principles according to the World Medical Association Declaration of Helsinki (2002 version) and was approved by the Institutional Review Board of Beijing Stomatological Hospital.

### Inclusion and Exclusion Criteria

To be included in this study, patients were required to fulfill the following criteria: (a) newly diagnosed HNSCC confirmed by pathology and no previous radiological or major surgical treatment; (b) a tumor located in the tongue, lower/upper gingiva, buccal mucosa, floor of the mouth, oropharynx or hard palate; (c) no evidence of distant metastasis; and (d) HNSCC with tumor stage II-IV according to UICC/AJCC classification 8^th^ edition.

The exclusion criteria were as follows: (a) patients who had unresectable disease at the time of surgery; (b) patients who refused major surgical treatment due to personal will; and (c) patients who underwent operation without free flap reconstruction.

### Goals and the Definition of Complications

The main goal of this study was to explore in-hospital complications and postoperative 42 days complications. POCs were defined as (a) postoperative respiratory or cardiac failure requiring critical care admission, (b) flap crisis, hematoma or any other complications requiring bedside treatment or reoperation, and (c) Surgical site infection (SSI) or pneumonia defined by the individual investigator or confirmed by bacterial cultivation. The length of hospital stay after the operation and total cost of hospital care were measured according to baseline records.

The Clavien-Dindo classification (CDC) is a widely accepted grading system based on an ordinal scale and demonstrated reliability for precisely classifying the severity of POCs (19). POCs were graded by the CDC system to classify severity: a minor complication was defined as grade I or II, while a severe complication was defined as grade III, IV or V (20) including death, life-threatening complications requiring Intensive Care/Intensive Care Unit (IC/ICU) management or complications requiring surgical, endoscopic or radiological intervention. The highest grade of POCs were recoded as the CDC grade of patients.

### Outcomes

The short-term outcomes included POCs, length of hospital stay after the operation and total cost of hospital care. The long-term outcomes were overall survival (OS) and disease-free survival (DFS). OS was calculated as the length of time from the first operation to all-cause death or the last follow‐up. DFS was defined as the length of time from the first operation until first recurrence, metastasis, or death. One-year and 2-year postoperative all-cause mortality were compiled with complete follow-up data.

## Covariates

Demographic factors (age, sex, Body Mass Index (BMI) and weight loss), tumor anatomy and pathological features (tumor site, T stage, pathological nodal [pN] stage, clinical features, and growth patterns) and operation‐related variables (operation time, blood loss, intraoperative fluid, tracheostomy[yes/no], type of flaps used, ND (unilateral/bilateral), type of antibiotic prophylaxis and red blood cell (RBC) transfusion during the operation[yes/no]) were recorded. Based on World Health Organization (WHO) cutoff points of BMI status, BMI were categorized into obese (≥30.0 kg/m^2^), overweight (25.0–29.9 kg/m^2^), normal weight (18.5–24.9 kg/m^2^), and underweight (<18.5 kg/m^2^) (14). Weight loss was defined as “weight loss >10% of the body weight within the past 6 months (21). Preoperative comorbidities (ACE-27 comorbidity index, hypertension, and diabetes) and habitual factors (smoking and alcohol histories) were collected and recorded through a person-to-person survey before surgery.

### Statistical Analyses

Baseline data are summarized as descriptive statistics. Categorical variables are presented as frequencies and percentages, and continuous variables are presented as the means ± standard deviations or medians (P25, P75). Univariate and multivariate logistic regression analyses were applied to explore risk factors for POCs and to build a forest plot. The odds ratios (ORs) with their 95% confidence intervals (CIs) and two‐tailed P values are reported. A prediction model that included all candidate predictors selected from the multivariate logistic regression analysis was built, and the results are presented as a nomogram. The concordance index (C-index) was used to determine discrimination ability of the nomogram. The area under the receiver operating characteristic curve (AUC) and ROC curve analysis were used to measure the difference between the predicted and observed outcomes. A calibration curve was adopted to compare the observed and predicted outcomes for the nomogram. Decision curve analysis (DCA) was used to test the predictive value of the model.

The survival curves were plotted by the Kaplan–Meier method to depict the associations of each group and the main outcome indexes, OS and DFS. Log‐rank tests were used to compare survival outcomes between different groups. The Cox proportional hazards regression model was used to assess the impacts of prognostic factors on DFS and OS. All tests were two‐sided, and P values <0.05 were considered statistically significant.

The data were analyzed with SPSS (version 17) and R software (version 4.0.4; https://www.R-project.org). The packages used included rms, pROC, rmda, forestplot, survival and survminer.

## Results

### Patient Characteristics

A total of 399 patients met the inclusion criteria: 250(62.7%) men and 149(37.3%) women. The mean patient age was 58.0 ± 10.7years. Of 399 patients, 155(38.8%) had complications in the perioperative period (from the day of the operation to 42 days after the operation). The results of the univariate logistic regression analysis showed that patients who experienced weight loss before the operation (P=0.021, OR 1.753, 95% [CI] 1.089-2.822) and those with a smoking history (P=0.009, OR 1.715, 95% [CI] 1.142-2.576), alcohol history (P=0.047, OR 1.509, 95% [CI] 1.006-2.264), ACE-27 index of 2-3(P=0.008, OR 2.446, 95% [CI] 1.268-4.721) and diabetes (P=0.047, OR 1.313, 95% [CI] 1.003-1.718) had a significantly higher risk of postoperative complications (**Table 1**).

**Table 1:** The univariate analysis between demographic and clinicopathological factors and POCs.

	Totaln = 399		POC (-) n = 244 (61.2%)	POC (+) n = 155 (38.8%)	P	OR (95% CI)
	No.	%	No. (%)	No. (%)		
Age					0.346	
>60	192	48.1	122 (50.0)	70 (45.2)		Ref.
≤60	207	51.9	122 (50.0)	85 (54.8)		1.214 (0.811-1.818)
Gender					0.300	
Male	250	62.7	148 (60.7)	102 (65.8)		Ref.
Female	149	37.3	96 (39.3)	53 (34.2)		0.801 (0.527-1.219)
BMI					0.807	
Underweight	9	2.2	6 (2.5)	3 (1.9)		Ref.
Normal	217	54.4	129 (52.9)	88 (56.8)		1.364 (0.332-5.600)
Overweight	152	38.1	97 (39.7)	55 (35.5)		1.134 (0.273-4.714)
Obese	21	5.3	12 (4.9)	9 (5.8)		1.500 (0.293-7.681)
Weight loss					0.021	
Absent	310	77.7	199 (81.6)	111 (71.6)		Ref.
Present	89	22.3	45 (18.4)	44 (28.4)		1.753 (1.089-2.822)
Smoking history					0.009	
Nonsmoker	200	50.4	135 (55.3)	65 (41.9)		Ref.
Smoker	199	49.6	109 (44.7)	90 (58.1)		1.715 (1.142-2.576)
Alcohol history					0.047	
Nondrinker	223	55.9	146 (59.8)	77 (49.7)		Ref.
Drinker	176	44.1	98 (40.2)	78 (50.3)		1.509 (1.006-2.264)
ACE-27					0.008	
0-1	358	89.6	227 (93.0)	131 (84.5)		Ref.
2-3	41	10.4	17 (7.0)	24 (15.5)		2.446 (1.268-4.721)
Hypertension					0.071	
Absent	251	62.9	162 (66.4)	89 (57.4)		Ref.
Present	148	37.1	82 (33.6)	66 (42.6)		1.465 (0.968-2.218)
Diabetes					0.047	
Absent	335	84.0	212 (86.9)	123 (79.4)		Ref.
Present	64	16.0	32 (13.1)	32 (20.6)		1.313 (1.003-1.718)
Tumor Sites					0.001	
Upper Gingiva	30	7.5	27 (11.1)	3 (1.9)		Ref.
Hard palate	9	2.3	7 (2.9)	2 (1.3)		
Tongue	153	38.3	97 (39.7)	56 (36.1)		4.857 (1.856-12.709)
Inferior gingiva	76	19.0	43 (17.6)	33 (21.3)		
Buccal Mucosa	73	18.3	39 (16.0)	34 (22.0)		
Floor of the mouth	43	10.8	21 (8.6)	22 (14.2)		
Oropharynx	15	3.8	10 (4.1)	5 (3.2)		
Growth Patterns					0.573	
Exophytic	99	24.8	64 (26.2)	35 (22.6)		Ref.
Ulcerative	153	38.3	89 (36.5)	64 (41.3)		1.315 (0.780-2.217)
Invasive	147	36.9	91 (37.3)	56 (36.1)		1.125 (0.663-1.911)
Clinical Stage					0.117	
II	87	21.8	61 (25.0)	26 (16.8)		Ref.
III	72	18.0	45 (18.4)	27 (17.4)		1.408 (0.726-2.729)
IV	240	60.2	138 (56.6)	102 (65.8)		1.734 (1.025-2.933)
T stage					0.006	
T2	103	25.8	65 (26.6)	38 (24.5)		Ref.
T3	99	24.8	65 (26.6)	34 (21.9)		
T4a	171	42.9	105 (43.0)	66 (42.6)		
T4b	26	6.5	9 (3.8)	17 (11.0)		3.217 (1.396-7.413)
pN stage					0.441	
N0	218	54.6	141 (57.8)	77 (49.7)		Ref.
pN1	68	17.1	38 (15.6)	30 (19.3)		1.446 (0.831-2.514)
pN2	89	22.3	52 (21.3)	37 (23.9)		1.303 (0.786-2.159)
pN3	24	6.0	13 (5.3)	11 (7.1)		1.549 (0.663-3.624)

POC, Postoperative complication.

BMI, body mass index.

ACE-27, Adult Comorbidity Evaluation-27 comorbidity index.

The most common primary tumor site was the tongue (153, 38.3%), followed by the inferior gingiva (76, 19.0%). According to postoperative pathological reports, the T stage was distributed as follows: T2 (n=103, 25.8%), T3 (n=99, 24.8%), T4a (n=171, 42.9%), and T4b (n=26, 6.5%); the lymph node status was distributed as follows: pN0 in 218(54.4%) patients, pN2 in 89 (22.3%), pN1 in 68 (17.0%), and pN3 in 25(6.3%). Tumor location in the non-upper gingiva and non-hard palate (Abbreviated as non-upper gingiva/hard palate) (P=0.001, OR 4.857, 95% [CI] 1.856-12.709) and T4b stage (P=0.006, OR 3.217, 95% [CI] 1.396-7.413) were significantly associated with POCs (**Table 1**).

The mean operation time and blood loss were 7.29 ± 1.44h and 611.02 ± 187.47ml in the POC (-) group and 8.00 ± 1.44h and 665.58 ± 230.27ml in the POC (+) group, with significant differences. Most patients received anterolateral thigh flaps (155, 38.8%), followed by radial forearm flaps (126, 31.6%), fibula flaps (106, 26.6%) and latissimus dorsi flaps (12, 3.0%). Cephalosporin was used to treat antibiotic prophylaxis in 376 patients, while clindamycin was used in the other 23 patients due to an allergy to cephalosporin. A total of 340 (85.2%) patients underwent unilateral ND, 50 (12.3%) underwent bilateral ND, and 9 (2.3%) did not undergo ND. In total, 271 (67.9%) patients underwent tracheostomy, and 18 (6.6%) of them had infectious pneumonia, significantly higher than those without tracheostomy (2/128, 1.6%) (P=0.028). Ninety-three (23.3%) patients received an RBC transfusion during the operation. An operation time≥8.0h (P=0.001, OR 2.584, 95% [CI] 1.706-3.915), blood loss>500 ml (P=0.034, OR 1.573, 95% [CI] 1.035-2.390), Clindamycin as antibiotic prophylaxis (vs. cephalosporin) (P=0.003, OR 3.897, 95% [CI] 1.565-9.707), bilateral ND (vs. unilateral or no ND) (P=0.043, OR 1.848, 95% [CI] 1.018-3.353) and tracheostomy (P=0.033, OR 1.622, 95% [CI] 1.040-2.530) were associated with an increased risk of POCs in univariate analysis (**Table 2**).

**Table 2:** The univariate analysis between operation‐related factors and POCs.

	Total n = 399		POC (-) n = 244 (61.2%)	POC (+) n = 155 (38.8%)	P	OR (95% CI)
	No.	%	No. (%)	No. (%)		
Operation time					0.001	
<8.0h	236	59.1	166 (68.0)	70 (45.2)		Ref.
≥8.0h	163	40.9	78 (32.0)	85 (54.8)		2.584 (1.706-3.915)
Blood loss					0.034	
0-500ml	160	40.1	108 (44.3)	52 (33.5)		Ref.
>500ml	239	59.9	136 (55.7)	103 (66.5)		1.573 (1.035-2.390)
Neck dissection					0.043	
None	9	2.3	7 (2.9)	2 (1.3)		Ref.
Unilateral	340	85.2	213 (87.3)	127 (81.9)		
Bilateral	50	12.5	24 (9.8)	26 (16.8)		1.848 (1.018-3.353)
Tracheostomy					0.033	
Absent	128	32.1	88 (36.1)	40 (25.8)		Ref.
Present	271	67.9	156 (63.9)	115 (74.2)		1.622 (1.040-2.530)
Flap Reconstruction					0.288	
Fibula flap	106	26.6	57 (23.4)	49 (31.6)		Ref.
Radial forearm flap	126	31.6	83 (34.0)	43 (27.8)		0.603 (0.355-1.024)
Anterolateral thigh flap	155	38.8	97 (39.7)	58 (37.4)		0.696 (0.421-1.149)
Latissimus dorsi flap	12	3.0	7 (2.9)	5 (3.2)		0.831 (0.248-2.785)
Antibiotic Prophylaxis					0.003	
Cephalosporin	376	94.2	237 (97.1)	139 (89.7)		Ref.
Clindamycin	23	5.8	7 (2.9)	16 (10.3)		3.897 (1.565-9.707)
Intraoperative Fluid					0.247	
<6000	318	79.7	199 (81.6)	119 (76.8)		Ref.
≥6000	81	20.3	45 (18.4)	36 (23.2)		1.338 (0.817-2.192)
RBC transfusion during operation					0.057	
No	306	76.7	195 (79.9)	111 (71.6)		Ref.
Yes	93	23.3	49 (20.1)	44 (28.4)		1.057 (0.987-2.522)

POC, Postoperative complication.

RBC, Red blood cell.

### Distributions of POCs and CDC Grades of Patients

The most common type of POC was SSI (95, 61.3%), followed by flap crisis or failure (37, 23.9%), pneumonia (20, 12.9%), hematoma (10, 6.4%), congestive heart failure (9, 5.8%), Acute Respiratory Distress Syndrome (ARDS) (6, 3.9%), Fistula (5, 3.2%), Cardio-discomfort (5, 3.2%), Pulmonary embolism (4, 2.6%), Airway-condition needs tracheotomy (4, 2.6%) and some other types.

Of all 155 patients with POCs, 88 (56.8%) patients were graded I or II in CDC, while 67 (43.2%) patients were graded III-V (**Supplemental Table 1**).

### Short-Term Outcomes

The median length of hospital stay after the operation in POC (+) patients was 14.00 (10.00, 21.00) days, which was significantly longer than that in POC (-) patients 9.00 (8.00, 11.75) days. The median healthcare cost in the POC (+) group was $6484.10 (5486.80,8162.90), whereas that in the POC (-) group was $5947.11 (4862.65,7081.87). A total of 137 (34.3%) patients received a transfusion while in the hospital. In total, 41.3% of patients in the POC (+) group received a transfusion, and 29.9% in the POC (-) group received a transfusion (**Table 3**).

**Table 3:** The association between post-operation complication and major short outcomes.

	Total n = 399	POC (-) n = 244 (61.2%)	POC (+) n = 155 (38.8%)	P
Cost	$6185.12 (5033.32,7527.23)	$5947.11 (4862.65,7081.87)	$6484.10 (5486.80,8162.90)	0.001
Length of hospital stay after operation (days)	10.00 (8.05,14.00)	9.00 (8.00,11.75)	14.00 (10.00,21.00)	0.001
RBC Transfusion				0.020
Absent	262 (65.7)	171 (70.1)	91 (58.7)	
Present	137 (34.3)	73 (29.9)	64 (41.3)	
1-year overall survival		93.6%	86.0%	0.015
Survive	327 (90.6)	204	123	
Death	34 (9.4)	14	20	
N/A	38	27	11	
2-year overall survival		84.5%	72.9%	0.024
Survive	217 (79.5)	131	86	
Death	56 (20.5)	24	32	
N/A	126	90	36	

POC, Postoperative complication.

RBC, Red blood cell.

### Multivariate Logistic Regression Analysis of POCs

**Figure 1:**
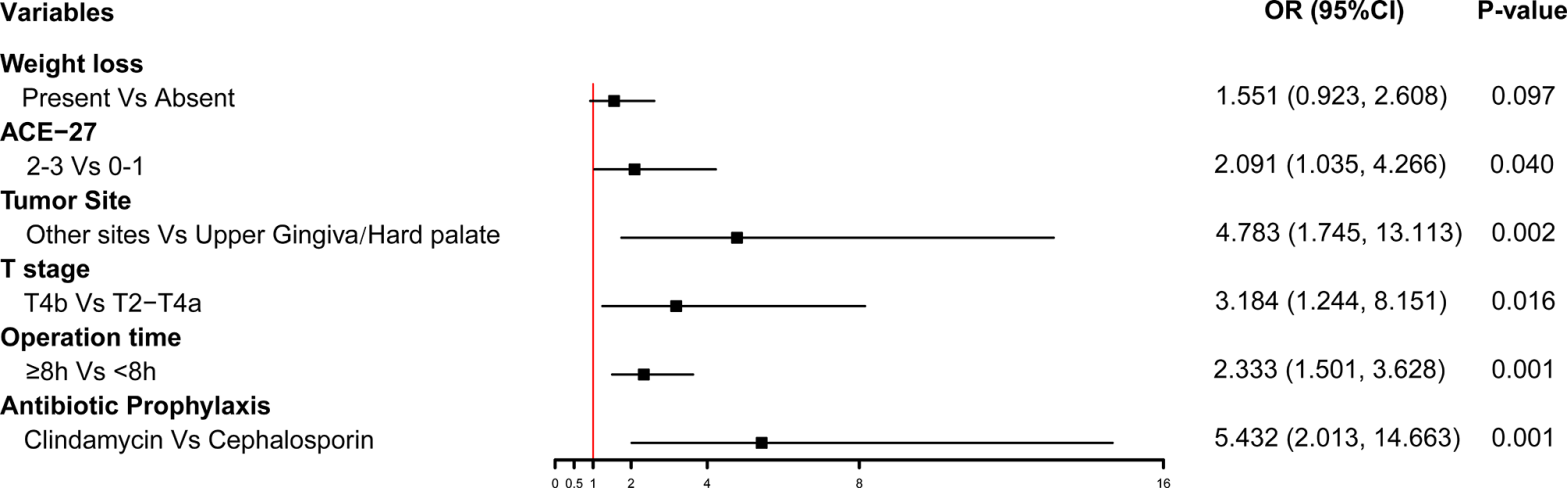
Multivariable logistic regression analysis was applied to build forest plots.

Multivariate logistic regression analysis on POCs showed that the independent risk factors were as follows: weight loss (P=0.097, OR 1.551, 95% [CI] 0.923-2.608), ACE-27 index:2-3 (vs. 0-1, P=0.040, OR 2.091, 95% [CI] 1.035-4.266), T4b stage (vs. T2-T4a, P=0.016, OR 3.184, 95% [CI] 1.244-8.151), tumor in the non-upper gingiva/hard palate (P=0.002, OR 4.783, 95% [CI] 1.745-13.113), operation time≥8h (P=0.001, OR 2.333, 95% [CI] 1.501-3.628), and Clindamycin as antibiotic prophylaxis (vs. cephalosporin, P=0.001, OR 5.432, 95% [CI] 2.013-14.663). The forest plot was built with the six variables (**Figure 1**).

### Development of a Novel Nomogram Prediction Model of POCs

**Figure 2:**
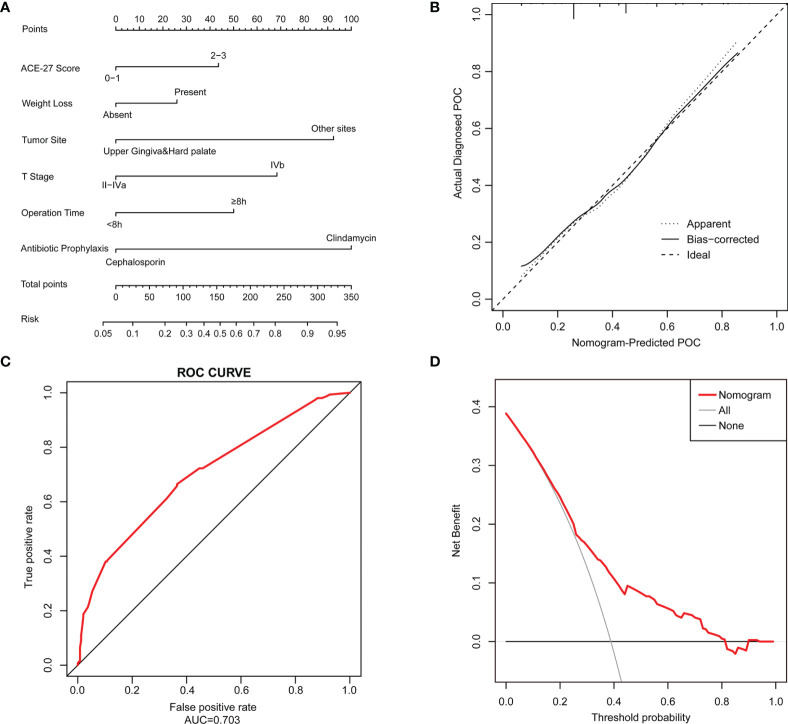
A Nomogram model is constructed to predict the POCs. **(A)** The POC risk nomogram was developed by incorporating the following factors: ACE-27 index, weight loss, tumor site, T stage of the tumor, operation time and type of antibiotic prophylaxis; **(B)** Calibration plots of the nomogram which the y-axis is the actual rate of POCs and the x-axis is the predicted rate of POCs. The diagonal dotted line represents a perfect prediction by an ideal model. The solid line represents the bias-corrected performance of the nomogram, where a closer fit to the diagonal dotted line represents a better prediction; **(C)** The accuracy of the model for identifying patients with POCs was determined using AUC curve; **(D)** DCA showed the clinical usefulness of the nomogram. The y-axis measures the net benefit. The red solid line is the nomogram used to predict POC risk. The gray solid line assumes that all patients will develop a POC. The thin black solid line assumes that no patients will develop a POC.

A nomogram that incorporated the six significant risk factors for predicting POCs was constructed (**Figure 2A**). The total score was calculated using the scores of the ACE-27 index, weight loss, tumor site, T stage, operation time and type of antibiotic prophylaxis.

The predictive nomogram achieved a C-index of 0.703, suggesting that the model has moderate discrimination ability. The calibration curve of the nomogram to predict POC risk after HNSCC surgery with free flap reconstruction demonstrated good consistency in this cohort (**Figure 2B**). The accuracies of the risk models were also compared using ROC curve analysis (AUC=0.703, **Figure 2C**).

DCA was used to determine whether the prediction model-based decisions were more clinically useful than default decisions for patients after surgery. The graph in **Figure 2D** shows the expected net benefit per patient to predict the risk of a POC when the nomogram score threshold was between 0.2-0.8 (red curve).

### Survival Analyses

Among the 399 patients in this study, 394(98.7%) had follow‐up data. The 1-year survival rates were 86.0% in the POC (+) group and 93.6% in the POC (-) group, and the 2-year survival rates were 72.9% and 84.5%, both with a significant difference.

**Figure 3:**
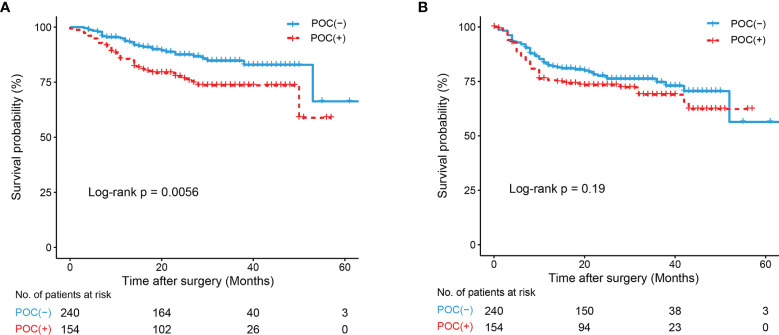
K-M curve drawn by occurrence of POCs and Overall survival (OS) and Disease-Free Survival (DFS) of all patients. **(A)** K-M curve of POCs and OS; **(B)** K-M curve of POCs and DFS.

Kaplan–Meier analysis revealed significant associations between POCs and OS (P<0.01, **Figure 3A**), and patients in the POC (-) group had a higher OS rate than those in the POC (+) group. No significant associations between POCs and DFS were observed (P=0.190, **Figure 3B**). We also found no significant difference in OS (P=0.841) or DFS (P=0.270) between patients with severe POCs and patients with minor POCs.

Among all the potential prognostic factors, T stage, N stage, hypertension, weight loss before the operation, operation time≥8.0h, RBC transfusion and POCs were all risk factors for OS (P<0.05) (**Table 4**). However, the association between POCs and OS was not significant after adjusting for other prognostic factors (P=0.128). Weight loss, N stage and RBC transfusion remained significant risk factors for OS (P<0.05). No operation-related factors were associated with a poor prognosis in the multivariate analysis of OS.

**Table 4:** Univariate & Multivariate Cox regression of risk factors for OS in all patients with HNSCC with free flap reconstruction.

	Univariate Cox regressionHR (95% CI)	P	Multivariate Cox regressionHR (95% CI)	P
Age		0.450		
>60	Ref.			
≤60	0.828 (0.507-1.352)			
Gender		0.732		
Male	Ref.			
Female	0.915 (0.549-1.524)			
BMI		0.333		
Underweight	Ref.			
Normal weight	1.184 (0.276-5.085)			
Overweight	0.890 (0.200-3.954)			
Obese	0.238 (0.021-2.610)			
Weight loss		0.001		0.001
Absent	Ref.		Ref.	
Present	3.899 (2.383-6.379)		3.255 (1.873-5.656)	
Smoking history		0.900		
Nonsmoker	Ref.			
Smoker	0.969 (0.594-1.582)			
Alcohol history		0.840		
Nondrinker	Ref.			
Drinker	0.950 (0.580-1.558)			
ACE-27		0.133		0.989
0-1	Ref.		Ref.	
2-3	1.648 (0.859-3.161)		0.995 (0.470-2.107)	
Hypertension		0.029		0.081
Absent	Ref.		Ref.	
Present	1.731 (1.059-2.828)		1.673 (0.938-2.984)	
Diabetes		0.149		0.669
Absent	Ref.		Ref.	
Present	1. 546 (0.855-2.797)		1.075 (0.771-1.499)	
Tumor Site		0.395		
Tongue	Ref.			
Inferior gingiva	1.274 (0.651-2.496)			
Buccal Mucosa	1.280 (0.647-2.532)			
Floor of the mouth	0.494 (0.147-1.663)			
Oropharynx	2.536 (0.951-6.760)			
Upper gingiva	1.300 (0.487-3.466)			
Hard palate	0.685 (0.092-5.110)			
Growth Patterns		0.731		
Exophytic	Ref.			
Ulcerative	1.216 (0.621-2.381)			
Invasive	1.309 (0.671-2.555)			
Clinical Stage		0.001		0.219
II	Ref.		Ref.	
III	0.695 (0.166-2.908)		0.232 (0.043-1.237)	
IV	4.600 (1.842-11.488)		0.555 (0.129-2.387)	
T stage		0.001		0.264
T2	Ref.		Ref.	
T3	1.811 (0.702-4.672)		1.968 (0.642-6.029)	
T4a	3.413 (1.515-7.690)		1.973 (0.630-6.182)	
T4b	6.434 (2.473-16.744)		3.629 (0.966-13.634)	
pN stage		0.001		0.001
N0	Ref.		Ref.	
N1	1.946 (0.891-4.253)		2.939 (1.162-7.433)	
N2	4.641 (2.506-8.596)		4.977 (2.230-11.105)	
N3	11.315 (5.258-24.350)		12.975 (4.907-34.311)	
Operation time		0.001		0.702
<8h	Ref.		Ref.	
≥8h	2.274 (1.373-3.766)		0.957 (0.762-1.201)	
Blood loss		0.098		0.448
0-500ml	Ref.		Ref.	
>500ml	1.573 (0.920-2.691)		0.764 (0.381-1.530)	
Neck resection		0.096		0.837
Unilateral	Ref.		Ref.	
Bilateral	1.711 (0.910-3.219)		0.914 (0.385-2.166)	
Tracheostomy		0.628		
Absent	Ref.			
Present	0.882 (0.529-1.468)			
Flap Reconstruction		0.158		
Fibula flap	Ref.			
Radial forearm flap	0.900 (0.464-1.747)			
Anterolateral thigh flap	1.185 (0.639-2.200)			
Latissimus dorsi flap	3.099 (1.028-9.340)			
Intraoperative Fluid		0.230		
<6000	Ref.			
≥6000	1.415 (0.803-2.493)			
RBC Transfusion		0.002		0.038
Absent	Ref.		Ref.	
Present	2.198 (1.345-3.592)		1.846 (1.033-3.298)	
POC		0.016		0.128
Negative	Ref.		Ref.	
Positive	1.944 (1.188-3.181)		1.519 (0.869-2.656)	

POC, Postoperative complication.

RBC, Red blood cell.

Ref., Reference group.

ACE-27, Adult Comorbidity Evaluation-27 comorbidity index.

## Discussion

The early postoperative period is a vulnerable time for HNSCC patients who have undergone a major operation with free flaps, as the risk of complications is increased. Reducing complications has become a major target for improving the quality of patient care and healthcare cost savings. A comprehensive analysis of the rates and types of complications is essential to develop appropriate interventions to reduce them.

Immediate flap reconstruction is generally associated with fewer POCs, a shorter hospital stay, reduced treatment costs and more favorable aesthetic and functional outcomes than cancer resection without flap reconstruction (22). However, reconstructive surgery using a microvascular free flap is also considered to be a great challenge, as it may lead to a longer operation time and more blood loss, both of which have been demonstrated to be associated with a high incidence of POCs (23, 24). Therefore, the significance of free flap reconstruction in the prognosis and occurrence of complications in HNSCC patients is complicated (25).

In the current study, we first developed a novel tool to predict the risk of POCs after HNSCC surgery with free flap reconstruction based on a prospective cohort with real-world data. Six parameters that may objectively reflect the risk of POCs were evaluated: preoperative factors (ACE-27 index and weight loss), tumor characteristics (T stage and tumor site) and perioperative factors (operation time and antibiotic prophylaxis). According to the validation, the predictive ability of our nomogram is reliable, and it can be widely used to predict POCs. However, considering that the limited number of patients may have influenced the veracity of our model, the nomogram was not externally validated.

Infective complications, including SSI and pneumonia, were the main types of POCs in HNSCC patients who underwent free flap reconstruction in this study. The probability of severe respiratory and circulatory complications is not high, which may have been due to strict preoperative preparation. While hair removal, antibiotic prophylaxis, the avoidance of hypothermia and perioperative glycemic control have been widely adopted to reduce infection-related complications (26), apart from a long surgery time and increased blood loss, ND and primary tumor site, which increase the risk of wound exposure to a microbacterial environment, can be high-level risk factors for SSI (27).

An increased risk of wound infections with flap reconstruction has been demonstrated (28, 29). Most clinicians agree that antibiotic prophylaxis is the most effective means to prevent infective complications (28, 30). According to American Society of Health-System Pharmacists (ASHP) guidelines (29, 31), cefazolin or cefuroxime with metronidazole, or ampicillin-sulbactam are recommended for oncological clean-contaminated head and neck surgery. The guidelines also suggest the use of clindamycin in patients with a beta-lactam allergy (29, 31). However, clindamycin may cause a 2-3 times higher risk of infective complications than beta-lactam in clean-contaminated head and neck cases (30, 32) and it was reported that no significant difference was observed between clindamycin and no antibiotic (33). Our study also demonstrated that prophylactic clindamycin led to a substantially higher risk of POCs than cephalosporin, suggesting that clindamycin is not sufficient and a broader antibiotic is needed.

An ever-expanding complex oncological surgery with free flap reconstruction often means a higher risk of massive intraoperative or postoperative hemorrhage, which may require a blood transfusion and lead to subsequent complications. Hemoglobin (Hb) was a critical indicator and the value below 7 g/dl was considered as a threshold for blood transfusion in head and neck surgical oncology according to guidelines (34), which was also accepted and used in our department. Moreover, the literature has demonstrated that blood transfusion may be considered as an important indicator for adverse short-term outcomes in patients undergoing oncological surgery (34, 35) and it was reported an almost 30% higher five-year overall survival rate of non-transfused OSCC patients than patients with transfusion (36). In this study, we found that 34.3% of patients required an RBC transfusion during the hospital stay, with a higher rate in the POC (+) group than in the POC (-) group. The univariate analysis also showed that patients who received a transfusion during surgery had a higher risk of POCs. Transfusion was also demonstrated to be a risk factor for long-term OS in the univariate and multivariate Cox regression analyses. Transfusion should be considered an important short-term outcome and a remarkable risk factor for long-term survival.

As maximum cytoreduction has been considered the ideal treatment for advanced HNSCC for decades, aggressive removal of the tumor to the greatest extent possible to improve survival is chosen by most oral and maxillofacial surgeons (37). Tumor characteristics are the main limitation of surgery for cancer patients (38). In our study, we found that not only long-term survival but also POC occurrence was highly associated with advanced T stage and N stage. A stage T4b tumor invades many important surrounding anatomical structures, such as the pterygoid plates, skull base or internal carotid artery, making complete excision of the tumor and hemostasis difficult and leading to a higher risk of POCs such as hematoma. A wide range of tumor resection may also result in surgical dead space and postoperative infections (26). Tumors in an advanced pN stage have extensive lymphatic metastasis or extranodal extension. Both of these are strongly associated with a poor prognosis.

A poor preoperative nutritional status in surgically treated patients may be an important factor affecting surgical tolerance and increasing the risk of complications (39, 40). Preoperative weight loss and BMI often reflect the nutritional status of patients. Preoperative weight loss may be more common in oral cancer (located at the beginning of the digestive tract) than in cancers at other locations because eating function is affected. In this study, preoperative weight loss occurred in 22.3% of patients, and it was identified as an important risk factor for POC occurrence and long-term survival. Preventing a decline in the nutritional status prior to surgery could be a means to reduce these negative consequences. In another study, our team found that the incidence of complications after OSCC surgery was highest (33.3%) in the low BMI group, but no significant relationship between BMI and POCs was demonstrated in this study (14). This may be due to the stricter screening procedure before free flap reconstruction for patients enrolled in this study.

A comorbidity assessment may be a crucial predictive factor for complications (41). The ACE-27 comorbidity index is a widely accepted comorbidity evaluation system for oncology patients. The ACE-27 index consists of 12 categories and 27 subcategories, each of which quantifies a specific disease within the circulatory, respiratory, digestive or nervous system and its severity (42). The index has been proven to be a validated, relevant scoring system for patients undergoing surgery for HNSCC (43, 44). Our study showed that an ACE-27 score>1 was significantly related to complications, which means that 2 or more comorbidities or a severe comorbidity can be a high-risk factor for poor short-term outcomes but not for long-term outcomes. Thus, the ACE-27 index can be useful when deciding which treatment option is more suitable for advanced-stage HNSCC patients.

It is well known that a prolonged operative time is often accompanied by a prolonged anesthesia time and more blood loss and may lead to many adverse events (45), such as SSI, wound disruption, reoperation or transfusion. A prolonged operative time was demonstrated to be associated with an increased risk of POCs in our studies. A long operation time increases wound exposure and decreases the effects of sterilization and antibacterial measures. It is generally believed that the operation time is closely related to the surgeon’s experience, the type of reconstruction, and a good preoperative design. Tracheostomy is a useful method to prevent asphyxia caused by airway obstruction after surgery. Direct exposure of the respiratory tract caused by tracheostomy may result in contamination, leading to adverse events such as pulmonary infection, which was demonstrated in our study. Tracheostomy tends to be used during tongue, floor of mouth or mandible resection, indicating a high risk of POCs.

Several limitations to this study need to be considered. Complications after complex surgery can never be completely eliminated and may have consequences that extend well beyond the postoperative period (38). Although based on a prospective real-world study, our study is still a single-center observational study with a limited sample size. However, the lost to follow-up rate of the research is very low, and the quality of prospective data can be well guaranteed. The avoidance of POCs remains a worthwhile goal, and further work is still needed to understand their occurrence. A multicenter prospective study with a large sample size may provide useful guidance for clinical decision making.

## Conclusion

The occurrence of POCs significantly increases the burden on patients and leads to poor long-term OS. More attention should be paid on operation time and blood loss. Measures should be taken to prevent weight loss before operation to reduce the risk of POCs. An antibiotic with a broader spectrum is better than clindamycin to prevent POCs.

## Data Availability Statement

The original contributions presented in the study are included in the article/**Supplementary Material**. Further inquiries can be directed to the corresponding author.

## Ethics Statement

The studies involving human participants were reviewed and approved by Institutional Review Board of Beijing Stomatological Hospital. The patients/participants provided their written informed consent to participate in this study.

## Author Contributions

DL: Project administration, validation, writing-original draft, interpretation of data. CW: Data analysis, writing-original draft, interpretation of data. BL: Validation, writing-original draft, interpretation of data. WW: Design of the work, interpretation of data, revising it critically for the intellectual content. HL, AC, QN: Resources, writing-original draft, interpretation of data. ZH: Supervision, writing-review & editing, resources. ZF: Funding acquisition, project administration, supervision, writing-original draft, interpretation of data. All authors contributed to the article and approved the final version.

## Funding

This article is supported by the Capital’s Funds for Health Improvement and Research (CFH2020-2-2143 and 2018-4-2082); the National Natural Science Foundation of China (82072984); the Project of Beijing Municipal Education Commission (KM202110025008); the Beijing Science and Technology Commission (Z161100000516201); the Discipline Construction Fund of Beijing Stomatological Hospital (18-09-21); and innovation Research Team Project of Beijing Stomatological Hospital, Capital Medical University, NO. CXTD202204.

## Conflict of Interest

The authors declare that the research was conducted in the absence of any commercial or financial relationships that could be construed as a potential conflict of interest.

## Publisher’s Note

All claims expressed in this article are solely those of the authors and do not necessarily represent those of their affiliated organizations, or those of the publisher, the editors and the reviewers. Any product that may be evaluated in this article, or claim that may be made by its manufacturer, is not guaranteed or endorsed by the publisher.
