# Supplementary material for: Postoperative Complications of Free Flap Reconstruction in Moderate-Advanced Head and Neck Squamous Cell Carcinoma: A Prospective Cohort Study Based on Real-World Data
Source: Front Oncol. 2022 Jun 24;12:792462. doi: 10.3389/fonc.2022.792462 (PMC9263716; doi:10.3389/fonc.2022.792462)
Supplement: Supplementary file 1 [file Table_1.docx]

Supplemental Table 1 CDC grades and the major POCs of patients

| CDC grade and major POC | No. | % of  POCs (+) | % of all patients |  |
| --- | --- | --- | --- | --- |
| **Grade I-II** | **88** | **56.8** | **22.1** |  |
| Surgical site infection | 69 | 44.5 | 17.3 |  |
| Pneumonia | 14 | 9.0 | 3.5 |  |
| Cardiac discomfort | 3 | 1.9 | 0.7 |  |
| Deliration | 1 | 0.7 | 0.3 |  |
| Others | 1 | 0.7 | 0.3 |  |
| **Grade III** | **54** | **34.8** | **13.5** |  |
| Flap cirsis |  |  |  |  |
| *Successfully saved* | 13 | 8.4 | 3.3 |  |
| *Failure* | 21 | 13.5 | 5.3 |  |
| Hematoma | 8 | 5.2 | 2.0 |  |
| Airway-condition needs tracheotomy | 4 | 2.6 | 1.0 |  |
| Surgical site infection | 3 | 1.9 | 0.7 |  |
| Fistula in surgical site | 3 | 1.9 | 0.7 |  |
| Others | 2 | 1.3 | 0.5 |  |
| **Grade IV** | **12** | **7.7** | **3.0** |  |
| CHF | 7 | 4.5 | 1.8 |  |
| PE | 3 | 1.9 | 0.7 |  |
| ARDS | 2 | 1.3 | 0.5 |  |
| **Grade V** | **1** | **0.7** | **0.3** |  |

**List of abbreviations:**

CDC: Clavien-Dindo classification POCs: Post-operation complications

CHF: Congestive heart failure ARDS: Acute respiratory distress syndrome

PE: Pulmonary embolism
