# Supplementary material for: Postoperative Complications of Free Flap Reconstruction in Moderate-Advanced Head and Neck Squamous Cell Carcinoma: A Prospective Cohort Study Based on Real-World Data
Source: Front Oncol. 2022 Jun 24;12:792462. doi: 10.3389/fonc.2022.792462 (PMC9263716; doi:10.3389/fonc.2022.792462)
Supplement: Supplementary file 2 [file Table_2.docx]

Supplemental Table 2 The multivariate analysis between risk factors and POCs

|  |  | POCs(+) | |
| --- | --- | --- | --- |
|  |  | OR (95% CI) | *P* |
| **Weight loss** |  |  | 0.097 |
| Absent |  | Ref. |  |
| Present |  | 1.511 (0.923 -2.608) |  |
| **ACE-27** |  |  | 0.040 |
| 0-1 |  | Ref. |  |
| 2-3 |  | 2.091 (1.035-4.266) |  |
| **Tumor site** |  |  | 0.002 |
| Absent |  | Ref. |  |
| Present |  | 4.783 (1.745-13.133) |  |
| **T stage** |  |  | 0.016 |
| T2-T4a |  | Ref. |  |
| T4b |  | 3.184 (1.244-8.151) |  |
| **Operation time** |  |  | 0.001 |
| <8.0h |  | Ref. |  |
| ≥8.0h |  | 2.333 (1.501-3.628) |  |
| **Antibiotic Prophylaxis** |  |  | 0.001 |
| Cephalosporin |  | Ref. |  |
| Clindamycin |  | 5.432 (2.013-14.633) |  |

**List of abbreviations:**

Ref.: Reference group

ACE-27: Adult Comorbidity Evaluation-27 comorbidity index
